# Association between lactate dehydrogenase and ventilator-associated pneumonia risk: an analysis of the MIMIC database 2001–2019

**DOI:** 10.1186/s12890-024-03084-9

**Published:** 2024-06-06

**Authors:** Xiangfeng Qian, Yi Sheng, Yinsheng Jiang, Yongan Xu

**Affiliations:** 1 Department of Emergency Medicine, Linping Campus, The Second Affiliated Hospital of Zhejiang University School of Medicine, Hangzhou, 311100 P.R. China; 2 Department of Emergency Medicine, The Second Affiliated Hospital of Zhejiang University School of Medicine, No. 88 Jiefang Road, Hangzhou, 310009 P.R. China

**Keywords:** Lactate dehydrogenase, Ventilator-associated pneumonia, Mechanical ventilation, Intensive care unit, MIMIC database

## Abstract

**Background:**

Serum lactate dehydrogenase (LDH) is a nonspecific inflammatory biomarker and has been reported to be associated with pneumonia prognosis. This study aimed to evaluate the relationship between LDH levels and ventilator-associated pneumonia (VAP) risk in intensive care unit (ICU) patients.

**Methods:**

This retrospective cohort study used data from the Multiparameter Intelligent Monitoring in Intensive Care database from 2001 to 2019. ICU patients aged ≥ 18 years and receiving mechanical ventilation were included. LDH levels were analyzed as continuous and categorical variables (< 210, 210–279, 279–390, > 390 IU/L), respectively. Restricted cubic spline (RCS) curves and quartiles were used to categorize LDH levels. Logistic regression and linear regression were utilized to assess the relationship of LDH levels with VAP risk and duration of mechanical ventilation, respectively.

**Results:**

A total of 9,164 patients were enrolled, of which 646 (7.05%) patients developed VAP. High levels of LDH increased the risk of VAP [odds ratio (OR) = 1.15, 95% confidence interval (CI): 1.06–1.24] and LDH levels were positively correlated with the duration of mechanical ventilation [β = 4.49, 95%CI: (3.42, 5.56)]. Moreover, patients with LDH levels of 279–390 IU/L (OR = 1.38, 95%CI: 1.08–1.76) and > 390 IU/L (OR = 1.50, 95%CI: 1.18–1.90) had a higher risk of VAP than patients with LDH levels < 210 IU/L. Patients with LDH levels of 279–390 IU/L [β = 3.84, 95%CI: (0.86, 6.82)] and > 390 IU/L [β = 11.22, 95%CI: (8.21, 14.22)] (vs. <210 IU/L) had a longer duration of mechanical ventilation.

**Conclusion:**

Elevated serum LDH levels were related to a higher risk of VAP and longer duration of mechanical ventilation and may be useful for monitoring VAP risk.

**Supplementary Information:**

The online version contains supplementary material available at 10.1186/s12890-024-03084-9.

## Introduction

Ventilator-associated pneumonia (VAP) is an infection of the lung parenchyma in patients exposed to invasive mechanical ventilation for at least 48 h [1]. VAP is one of the most common acquired pneumonias in intensive care unit (ICU) patients, affecting 5-40% of patients receiving invasive mechanical ventilation [1–3]. VAP is associated with increased length of hospitalization, mortality, infections caused by multidrug-resistant pathogens, and a heavy economic burden [4–6]. A meta-analysis based on randomized controlled studies showed that the overall attributable mortality rate for VAP was 13%, with higher mortality rates in surgical patients and those with a moderate severity score on admission [7]. Identifying risk factors associated with AVP is important for disease prevention and management.

The endotracheal tube is one of the major risk factors for VAP because it may serve as a reservoir for potentially infectious microorganisms, while at the same time it can bypass host defenses and act as a bridge between the oropharyngeal environment and the sterile bronchoalveolar space [8]. Other risk factors associated with the development of VAP include duration of mechanical ventilation, chronic lung disease, acute respiratory distress, sepsis, red blood cell transfusions, neurologic disorders, and prior antibiotic use [9]. Recently, several studies have reported a relationship between lactate dehydrogenase (LDH) levels and other pneumonia [10–12]. LDH levels can be used as a biomarker to predict refractory Mycoplasma pneumoniae pneumonia in early hospitalization [10]. Elevated LDH levels were related to an increased risk of acquired pneumonia [11]. Serum LDH is a metabolic and prognostic biomarker as well as a nonspecific biomarker of inflammation, and high LDH levels are usually associated with poor prognosis [13]. However, the relationship between LDH levels and VAP risk remains unclear. Herein, this study aimed to explore the relationship between LDH levels and VAP risk in ICU patients to provide more evidence for the monitoring of VAP risk.

## Methods

### Study design and population

Data for this retrospective cohort study were obtained from the Multiparameter Intelligent Monitoring in Intensive Care III and IV (MIMIC-III/IV) database from 2001 to 2019 (https://mimic.mit.edu/docs/iii/). MIMIC-III is a large, publicly available database that collects hospitalization data for more than 40,000 patients admitted to the ICU at Beth Israel Deaconess Medical Center between 2001 and 2012. MIMIC-IV is an updated MIMIC-III and collects hospitalization data for ICU patients between 2008 and 2019. The MIMIC database collects patient demographics, interventions, medical history, clinical measures, clinical laboratory tests, and medical data. Patients were included according to the following criteria: (1) patients who received mechanical ventilation; (2) patients aged ≥ 18 years; and (3) patients who were admitted to the ICU for more than 48 h. Patients with missing LDH levels on the first admission were excluded. MIMIC-III database was approved by the Institutional Review Boards of Beth Israel Deaconess Medical Center and the Massachusetts Institute of Technology, and informed consent was obtained from each patient. In this study, local institutional review board approval and informed consent were not required because MIMIC research data were publicly available and all patient data were de-identified.

### Outcomes

The outcome of this study was the occurrence of VAP. Patients with VAP in the MIMIC database were identified based on the International Classification of Diseases 9th edition and 10th edition codes (ICD-9: 4957 and 99,731; ICD-10: J95851). The length of follow-up was from the time the patient was admitted to the ICU to the time the patient was discharged from the hospital or had a VAP. For patients with multiple ICU admissions recorded, only data from the patient’s first ICU admission were analyzed.

### Data collection

Patient data were collected including age, gender (male, female), insurance (Medicare, others), weight, heart rate, systolic blood pressure (SBP), diastolic blood pressure (DBP), respiratory rate, temperature, saturation of peripheral oxygen (SPO_2_), white blood cell (WBC), international normalized ratio (INR), prothrombin time, vasopressors, antibiotics, sepsis, Sequential Organ Failure Assessment (SOFA) score, Simplified Acute Physiology Score (SAPS) II, Glasgow Coma Score (GCS), Charlson comorbidity index (CCI), myocardial infarction, congestive heart failure, liver disease, malignant cancer, pneumonia, chronic obstructive pulmonary disease (COPD), acute kidney injury (AKI), acute respiratory distress syndrome (ARDS), type of mechanical ventilation (invasive, non-invasive), duration of mechanical ventilation, and LDH levels at admission.

### Statistical analysis

Continuous data were presented as median and quartiles [M (Q1, Q3)], and the Mann-Whitney U test was applied to compare differences between groups. Categorical data were presented as numbers and percentages [n (%)], and the Chi-square test or Fisher’s exact test was applied to compare differences between groups.

Confounders associated with VAP risk were screened using the univariable logistic regression model (Supplementary Table S1), and variables with *P* < 0.05 were included in the multivariable regression models for adjustment (model 2). In addition, a Directed Acyclic Graph (DAG) was applied to elucidate the associations of confounders with LDH and VAP. Potential confounders presented in the DAG were also adjusted for in the multivariate regression models (model 3). The effects of confounders on VAP and duration of mechanical ventilation were presented in Supplementary Tables S2 and S3. The univariable and multivariable logistic regression models were utilized to explore the relationship between LDH levels and VAP risk, and results were presented as odds ratio (OR) and 95% confidence interval (CI). LDH levels were analyzed as continuous and categorical variables, respectively. Restricted cubic spline (RCS) curves were utilized to explore the relationship between LDH levels and VAP risk. LDH levels were categorized into four groups [< Q1 (< 210 IU/L), Q1-cutoff value (210–279 IU/L), cutoff value-Q3 (279–390 IU/L), > Q3 (> 390 IU/L)] based on the quartiles of LDH and the cutoff values of the RCS curves. The association between LDH levels and the risk of VAP was stratified according to type of mechanical ventilation (invasive, non-invasive), duration of mechanical ventilation (≤ 27.2 h, > 27.2 h), sepsis (no, yes), and antibiotics (no, yes). Moreover, the linear regression models were utilized to analyze the relationship between LDH levels and duration of mechanical ventilation, and results were expressed as beta values (β) and 95%CI. Statistical analyses were conducted using R 4.2.0 software (Institute for Statistics and Mathematics, Vienna, Austria). A P-value < 0.05 was considered statistically significant.

## Results

### Characteristics of patients

**Fig. 1:**
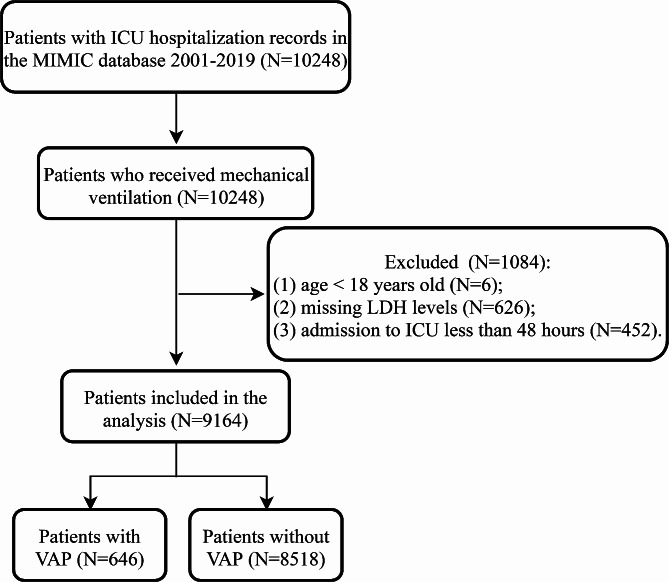
The flow chart of the study population. MIMIC, the Multiparameter Intelligent Monitoring in Intensive Care database; ICU, intensive care unit; LDH, lactate dehydrogenase; VAP, ventilator-associated pneumonia

A total of 10,248 patients who received mechanical ventilation were recorded in the MIMIC database between 2001 and 2019. After screening, 1,084 patients were excluded and 9,164 patients were included in the analysis (Fig. 1). The characteristics of these 9,164 patients were listed in Table 1. The median age of patients was 65.0 (53.7, 77.0) years and 5,189 (56.6%) patients were male. The median duration of mechanical ventilation was 27.2 (12.0, 56.0) hours and 4,495 (49.1%) patients received invasive mechanical ventilation. There were 4,389 (47.9%) patients who received vasopressors and 6,948 (75.8%) patients received antibiotics. The median LDH levels were 279.0 (210.0, 390.0) IU/L. There were 646 (7.0%) patients who developed VAP and 8,518 (93.0%) patients who did not.

**Table 1:** Characteristics of patients receiving mechanical ventilation

Variable	Total (*n* = 9164)	Non-VAP (*n* = 8518)	VAP (*n* = 646)	*P*
Age, years, M (Q_1_, Q_3_)	65.0 (53.7, 77.0)	65.8 (54.0, 77.0)	61 (50.0, 73.0)	< 0.001
Gender, n (%)				< 0.001
Female	3975 (43.4)	3755 (44.1)	220 (34.1)	
Male	5189 (56.6)	4763 (55.9)	426 (65.9)	
Insurance, n (%)				0.006
Medicare	4489 (49.0)	4206 (49.4)	283 (43.8)	
Others	4675 (51.0)	4312 (50.6)	363 (56.2)	
Weight, kg, M (Q_1_, Q_3_)	79.8 (66.5, 95.2)	79.3 (66.4, 95)	84.3 (68.9, 99)	< 0.001
Heart Rate, bpm, M (Q_1_, Q_3_)	91.0 (77.0, 107.0)	91.0 (77.0, 107.0)	92.0 (78.0, 108.0)	0.389
SBP, mmHg, M (Q_1_, Q_3_)	120.0 (104.0, 139.0)	119.0 (104.0, 139.0)	122.0 (105.0, 140.0)	0.101
DBP, mmHg, M (Q_1_, Q_3_)	66.0 (55.0, 78.0)	66.0 (55.0, 78.0)	68.0 (56.0, 81.0)	0.004
Respiratory rate, bpm, M (Q_1_, Q_3_)	20.0 (16.0, 24.0)	20.0 (16.0, 24.0)	19.0 (16.0, 24.0)	0.672
Temperature, ℃, M (Q_1_, Q_3_)	36.7 (36.3, 37.2)	36.7 (36.3, 37.2)	36.8 (36.3, 37.2)	0.213
Spo2, %, M (Q_1_, Q_3_)	98.0 (95.0, 100.0)	98.0 (95.0, 100.0)	98.0 (94.0, 100.0)	0.253
SOFA, M (Q_1_, Q_3_)	2.0 (1.0, 4.0)	2.0 (1.0, 4.0)	3.0 (1.0, 5.0)	< 0.001
SAPSII, M (Q_1_, Q_3_)	39.0 (31.0, 50.0)	39.0 (31.0, 49.0)	43.0 (32.0, 52.0)	< 0.001
GCS, M (Q_1_, Q_3_)	15.0 (15.0, 15.0)	15.0 (15.0, 15.0)	15.0 (15.0, 15.0)	< 0.001
CCI, M (Q_1_, Q_3_)	3.0 (2.0, 6.0)	3.0 (2.0, 6.0)	3.0 (2.0, 5.0)	0.044
WBC, K/uL, M (Q_1_, Q_3_)	11.5 (7.9, 16.2)	11.4 (7.9, 16.2)	11.8 (8.9, 16.3)	0.026
INR, M (Q_1_, Q_3_)	1.3 (1.2, 1.6)	1.3 (1.2, 1.6)	1.3 (1.1, 1.6)	0.537
PT, seconds, M (Q_1_, Q_3_)	14.6 (13.0, 17.7)	14.6 (13.0, 17.7)	14.4 (12.9, 17.9)	0.612
Type of mechanical ventilation, n (%)				< 0.001
Invasive mechanical ventilation	4495 (49.1)	3979 (46.7)	516 (79.9)	
Non-Invasive mechanical ventilation	4669 (50.9)	4539 (53.3)	130 (20.1)	
Duration, hours, M (Q_1_, Q_3_)	27.2 (12.0, 56.0)	26.2 (11.9, 54.2)	40.3 (15.0, 103.1)	< 0.001
Vasopressors, n (%)				< 0.001
No	4775 (52.1)	4510 (52.9)	265 (41.0)	
Yes	4389 (47.9)	4008 (47.1)	381 (59.0)	
Antibiotics, n (%)				0.016
No	2216 (24.2)	2085 (24.5)	131 (20.3)	
Yes	6948 (75.8)	6433 (75.5)	515 (79.7)	
Sepsis, n (%)				< 0.001
No	6617 (72.2)	6234 (73.2)	383 (59.3)	
Yes	2547 (27.8)	2284 (26.8)	263 (40.7)	
Myocardial infarction, n (%)				0.231
No	7441 (81.2)	6905 (81.1)	536 (83.0)	
Yes	1723 (18.8)	1613 (18.9)	110 (17.0)	
Congestive heart failure, n (%)				0.490
No	6000 (65.5)	5569 (65.4)	431 (66.7)	
Yes	3164 (34.5)	2949 (34.6)	215 (33.3)	
Liver disease, n (%)				< 0.001
No	7340 (80.1)	6858 (80.5)	482 (74.6)	
Yes	1824 (19.9)	1660 (19.5)	164 (25.4)	
Malignant cancer, n (%)				< 0.001
No	7814 (85.3)	7229 (84.9)	585 (90.6)	
Yes	1350 (14.7)	1289 (15.1)	61 (9.4)	
Pneumonia, n (%)				0.119
No	6755 (73.7)	6262 (73.5)	493 (76.3)	
Yes	2409 (26.3)	2256 (26.5)	153 (23.7)	
COPD, n (%)				0.645
No	7183 (78.4)	6672 (78.3)	511 (79.1)	
Yes	1981 (21.6)	1846 (21.7)	135 (20.9)	
AKI, n (%)				< 0.001
No	1554 (17.0)	1525 (17.9)	29 (4.5)	
Yes	7610 (83.0)	6993 (82.1)	617 (95.5)	
ARDS, n (%)				< 0.001
No	7658 (83.6)	7187 (84.4)	471 (72.9)	
Yes	1506 (16.4)	1331 (15.6)	175 (27.1)	
LDH, IU/L, M (Q_1_, Q_3_)	279.0 (210.0, 390.0)	276.0 (209.0, 386.0)	308.5 (228.0, 454.0)	< 0.001
LDH, n (%)				< 0.001
< 210 IU/L	2314 (25.3)	2193 (25.7)	121 (18.7)	
210–279 IU/L	2290 (25.0)	2153 (25.3)	137 (21.2)	
279–390 IU/L	2275 (24.8)	2100 (24.7)	175 (27.1)	
> 390 IU/L	2285 (24.9)	2072 (24.3)	213 (33.0)	

Note VAP, ventilator-associated pneumonia; SBP, systolic blood pressure; DBP, diastolic blood pressure; SPO_2_, saturation of peripheral oxygen; SOFA, Sequential Organ Failure Assessment score; SAPSII, Simplified Acute Physiology Score II; GCS, Glasgow Coma Score; CCI, Charlson comorbidity index, WBC, white blood cell; INR, international normalized ratio; PT, prothrombin time; COPD, chronic obstructive pulmonary disease; AKI, acute kidney injury; ARDS, acute respiratory distress syndrome; LDH, lactate dehydrogenase

### Relationship between LDH levels and VAP risk and duration of mechanical ventilation

**Fig. 2:**
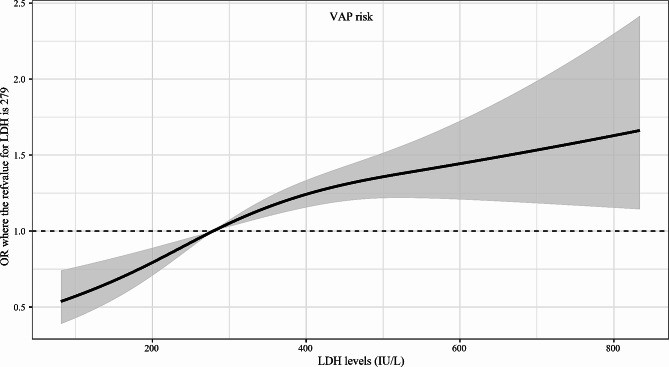
The restricted cubic spline (RCS) curves between lactate dehydrogenase (LDH) levels and ventilator-associated pneumonia (VAP) risk. OR, odds ratio

The RCS curves showed that the risk of VAP increased with the increase of LDH levels (Fig. 2). The incidence of VAP and duration of mechanical ventilation in the four LDH level groups was presented in Supplementary Figure S1. The incidence of VAP and duration of mechanical ventilation increased in stages in the group with higher LDH levels.

**Fig. 3:**
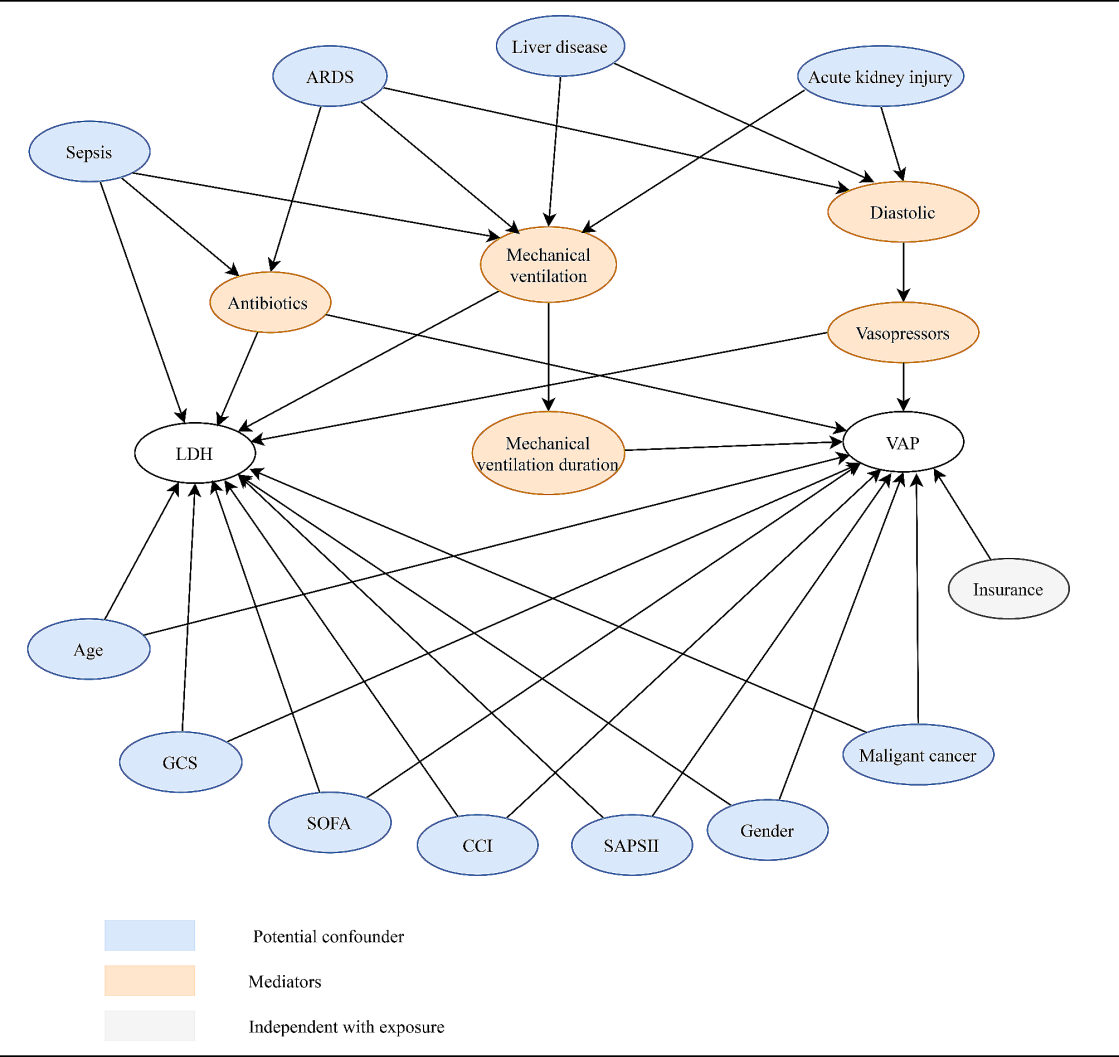
A Directed Acyclic Graph (DAG) for elucidating the associations of confounders with lactate dehydrogenase (LDH) levels and ventilator-associated pneumonia (VAP). Variables associated with both LDH and VAP (potential confounders) were adjusted for in multivariable analyses, whereas mediators were not adjusted. SOFA, Sequential Organ Failure Assessment score; SAPSII, Simplified Acute Physiology Score II; GCS, Glasgow Coma Score; CCI, Charlson comorbidity index, ARDS, acute respiratory distress syndrome

The relationship between LDH levels and VAP risk was showed in Table 2. The results demonstrated that high levels of LDH increased the risk of VAP (OR = 1.24, 95%CI: 1.16–1.34) when LDH was used as a continuous variable. After adjusting for age, gender, insurance, sepsis, DBP, SOFA, SAPS II, GCS, CCI, type of mechanical ventilation, duration of mechanical ventilation, vasopressors, antibiotics, liver disease, malignant cancer, AKI, and ARDS (model 2), high levels of LDH increased the risk of VAP (OR = 1.12, 95%CI: 1.03–1.21). After adjustment for confounders in DAG (model 3: age, gender, sepsis, SOFA, SAPS II, GCS, CCI, liver disease, malignant cancer, AKI, and ARDS) (Fig. 3), high levels of LDH were still associated with a higher risk of VAP (OR = 1.15, 95%CI: 1.06–1.24). When LDH was used as a categorical variable, patients with LDH levels of 279–390 IU/L (OR = 1.51, 95%CI: 1.19–1.92) and > 390 IU/L (OR = 1.86, 95%CI: 1.48–2.35) had a higher risk of VAP than patients with LDH levels < 210 IU/L in univariable analysis, but not in patients with LDH levels of 210–279 IU/L (*P* = 0.267). Moreover, patients with LDH levels of 279–390 IU/L (OR = 1.38, 95%CI: 1.08–1.76) and > 390 IU/L (OR = 1.50, 95%CI: 1.18–1.90) still had a higher risk of VAP than patients with LDH levels < 210 IU/L after adjustment for confounders (model 3).

**Table 2:** The relationship between lactate dehydrogenase (LDH) levels and ventilator-associated pneumonia (VAP) risk

Exposures	Model 1		Model 2		Model 3	
	OR (95%CI)	*P*	OR (95%CI)	*P*	OR (95%CI)	*P*
LDH levels	1.24 (1.16–1.34)	< 0.001	1.12 (1.03–1.21)	0.007	1.15 (1.06–1.24)	< 0.001
**LDH levels**						
<210 IU/L	Ref	-	Ref	-	Ref	-
210–279 IU/L	1.15 (0.90–1.48)	0.267	1.03 (0.79–1.33)	0.847	1.09 (0.84–1.40)	0.520
279–390 IU/L	1.51 (1.19–1.92)	< 0.001	1.23 (0.96–1.58)	0.100	1.38 (1.08–1.76)	0.010
>390 IU/L	1.86 (1.48–2.35)	< 0.001	1.34 (1.05–1.71)	0.019	1.50 (1.18–1.90)	< 0.001

Note OR, odds ratio; CI, confidence interval; Ref, reference;

Model 1 was univariable logistic regression analysis;

Model 2 was multivariable logistic regression analysis adjusted for age, gender, insurance, sepsis, DBP, SOFA, SAPS II, GCS, CCI, type of mechanical ventilation, duration of mechanical ventilation, vasopressors, antibiotics, liver disease, malignant cancer, AKI, and ARDS

Model 3 was multivariable logistic regression analysis adjusted for age, gender, sepsis, SOFA, SAPS II, GCS, CCI, liver disease, malignant cancer, AKI, and ARDS

**Fig. 4:**
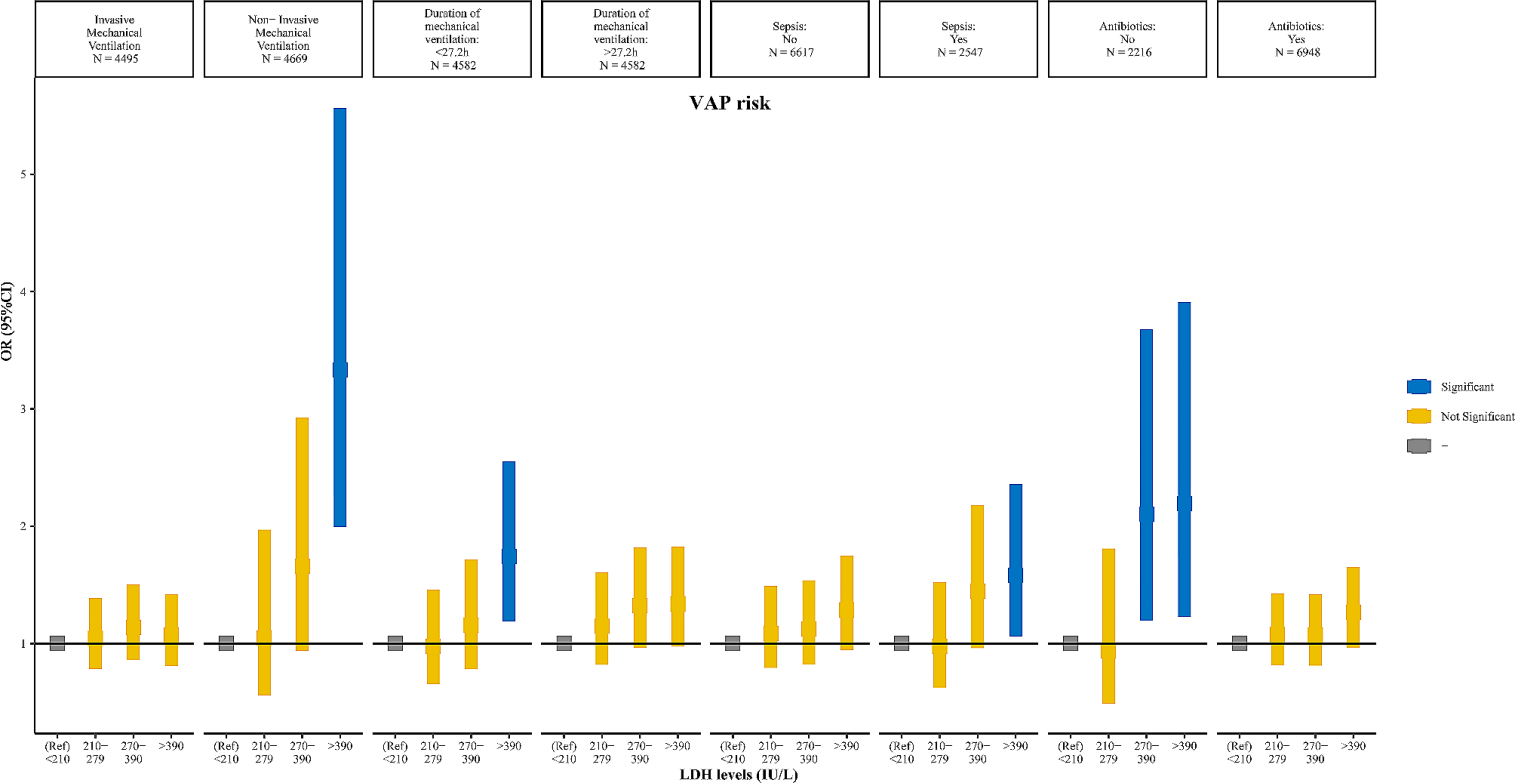
Stratified analysis of the association between lactate dehydrogenase (LDH) levels and ventilator-associated pneumonia (VAP) risk. OR, odds ratio; CI, confidence interval. All analyses were multivariable logistic regression analysis adjusted for age, gender, insurance, sepsis, DBP, SOFA, SAPS II, GCS, CCI, type of mechanical ventilation, duration of mechanical ventilation, vasopressors, and antibiotics

The results of the stratified analysis between LDH levels and VAP risk were shown in Fig. 4. In patients with different types of mechanical ventilation, only non-invasive mechanical ventilation patients with LDH levels > 390 IU/L (vs. <210 IU/L) (*P* < 0.05) had a higher risk of VAP. Among patients with a duration of mechanical ventilation < 27.2 h, the risk of VAP was higher in patients with LDH levels > 390 IU/L (vs. <210 IU/L) (*P* < 0.05). Patients with sepsis who had LDH levels > 390 IU/L (vs. <210 IU/L) (*P* < 0.05) were at increased risk for VAP. Moreover, patients who did not receive antibiotics had an increased risk of VAP at LDH levels of 279–390 IU/L and > 390 IU/L (*P* < 0.05) (vs. <210 IU/L) increased the risk of VAP.

The relationships between LDH levels and the duration of mechanical ventilation were presented in Table 3. LDH levels were positively correlated with the duration of mechanical ventilation in univariable [β = 6.60, 95%CI: (5.52, 7.67)] and multivariable [β = 4.49, 95%CI: (3.42, 5.56)] analyses when LDH was used as a continuous variable. When LDH was used as a categorical variable, patients with LDH levels of 279–390 IU/L [β = 3.84, 95%CI: (0.86, 6.82)] and > 390 IU/L [β = 11.22, 95%CI: (8.21, 14.22)] (vs. <210 IU/L) had a longer duration of mechanical ventilation in multivariable analysis.

**Table 3:** The relationship between lactate dehydrogenase (LDH) levels and duration of mechanical ventilation

Exposures	Model 1		Model 2		Model 3	
	Beta (95%CI)	*P*	Beta (95%CI)	*P*	Beta (95%CI)	*P*
LDH levels	6.60 (5.52, 7.67)	< 0.001	4.64 (3.57, 5.70)	< 0.001	4.49 (3.42, 5.56)	< 0.001
**LDH levels**						
<210 IU/L	Ref	-	Ref	-	Ref	-
210–279 IU/L	2.33 (-0.70, 5.36)	0.131	0.66 (-2.29, 3.61)	0.663	1.21 (-1.76, 4.17)	0.425
279–390 IU/L	6.55 (3.52, 9.59)	< 0.001	3.24 (0.27, 6.20)	0.032	3.84 (0.86, 6.82)	0.011
>390 IU/L	16.61 (13.58, 19.64)	< 0.001	11.18 (8.19, 14.17)	< 0.001	11.22 (8.21, 14.22)	< 0.001

Note CI, confidence interval; Ref, reference;

Model 1 was univariable linear regression analysis;

Model 2 was multivariable linear regression analysis adjusted for age, gender, insurance, sepsis, DBP, SOFA, SAPS II, GCS, CCI, type of mechanical ventilation, duration of mechanical ventilation, vasopressors, antibiotics, liver disease, malignant cancer, AKI, and ARDS.

Model 3 was multivariable linear regression analysis adjusted for age, gender, sepsis, SOFA, SAPS II, GCS, CCI, liver disease, malignant cancer, AKI, and ARDS

## Discussion

This study examined the association between LDH levels and the risk of VAP in patients receiving mechanical ventilation. High LDH levels increased the risk of VAP in patients with mechanical ventilation. Moreover, high LDH levels were associated with a longer duration of mechanical ventilation.

VAP is one of the most common acquired infections in ICU patients [1]. Mechanical ventilation damages the tracheal epithelium, resulting in increased infection by environmental microorganisms and migration of environmental microorganisms in the respiratory tract [14]. Microaspiration and biofilm formation are the two most important mechanisms by which VAP occurs [8]. Microaspiration occurs when microorganisms in secretions that accumulate above the endotracheal tube cuff migrate distally [15]. Secretions and attached microorganisms form a network, or biofilm, that migrates along the endotracheal tube cuff aggregates and inside the lumen of the endotracheal tube, thereby facilitating transfer to the sterile bronchial tree [16]. Oropharyngeal contents may be introduced into the airway during endotracheal intubation, and some studies have shown that bacteria have been observed to colonize endotracheal tubes after several hours of mechanical ventilation [17]. LDH is a widespread enzyme and a nonspecific biomarker of inflammation, and LDH levels may be elevated during inflammatory processes [10]. Several studies have reported the associations of high LDH levels with acquired pneumonia, mycoplasma pneumoniae pneumonia, and complicated pneumonia [10–12]. High LDH levels increase the risk of death in patients with acquired pneumonia [11]. Serum LDH is a significant factor in predicting mycoplasma pneumoniae pneumonia, and the area under the curve of the LDH prediction effect reaches 0.718 [10]. Elevated LDH levels are significantly associated with prolonged hospitalization in patients with complicated pneumonia [12]. Our results demonstrated that elevated LDH levels increased the risk of VAP in patients with mechanical ventilation, which was consistent with previous findings in the association between LDH and mycoplasma pneumoniae pneumonia [10]. In addition, our results showed that high LDH levels were related to a longer duration of mechanical ventilation. Duration of mechanical ventilation is a risk factor for the development of VAP [18]. However, the relationship between serum LDH levels and duration of mechanical ventilation has rarely been reported in previous studies. In conclusion, elevated LDH levels in patients undergoing mechanical ventilation may suggest a high risk of VAP, which may prompt clinicians to intervene promptly to reduce the burden of VAP.

The effect of LDH levels on VAP risk may be related to the function of LDH. Elevated LDH levels reflect tissue or cellular damage and are considered a common sign of tissue or cellular injury, suggestive of viral infection or lung injury [19, 20]. Severe infections may result in cytokine-mediated tissue damage and LDH release [21]. Park et al. showed that pulmonary LDH released during infection is an important target for streptococcus pneumoniae binding via pneumococcal surface protein A and pneumococcal surface protein C, and that pneumococci benefit in vivo by utilizing LDH-A-derived lactic acid [22]. Moreover, serum LDH is a prognostic biomarker for metabolism and immunomonitoring, and elevated serum LDH is associated with immunocompromise [23, 24]. LDH promotes lactate synthesis, which enhances immunosuppressive cells such as macrophages, and suppresses cytolytic cells such as NK cells and cytotoxic T lymphocytes [23]. Low LDH activity provides anti-inflammatory effects by down-regulating various inflammatory mediators (e.g., cytokines, NO) as well as lactate fluctuations that modulate macrophage inflammatory responses [25]. Elevated LDH may lead to hyperactivation of lung tissue inflammation and reduced immune response, all of which are associated with a poor prognosis in patients with severe infections [26, 27]. These roles of LDH may explain the association between elevated LDH levels and increased risk of VAP. However, additional mechanisms of the effect of serum LDH levels on VAP may need to be explored in more studies.

We analyzed the relationship between LDH levels and the risk of VAP in ICU patients, which may provide additional evidence for the recognition of VAP risk factors. Nevertheless, several limitations should be considered. First, the data in the MIMIC database are from a single center, and future studies need multicenter prospective data. Second, we only analyzed the relationship between LDH levels at initial ICU admission and the risk of VAP, whereas the effect of changes in LDH levels after admission to ICU on the risk of VAP is still unclear. Third, the effect of LDH levels on the risk of different types of VAPs is unclear due to database limitations. Fourth, we can only obtain whether patients have VAP after admission to the ICU, but cannot obtain the specific time of VAP occurrence due to the limitations of the MIMIC database.

## Conclusions

This study assessed the relationship between LDH levels and the risk of VAP in ICU patients based on a large sample MIMIC database. Elevated serum LDH levels were associated with a higher risk of VAP and a longer duration of mechanical ventilation. Serum LDH levels may be useful as a biomarker for VAP risk monitoring. Future studies may need to investigate the effect of changes in LDH levels on VAP risk based on prospective data.

### Electronic supplementary material

Below is the link to the electronic supplementary material.

Supplementary Material 1

## Data Availability

The datasets used and/or analyzed during the current study are available from the MIMIC-III and -IV database, https://mimic.mit.edu/docs/iii/, https://mimic.physionet.org/iv/.

## Abbreviations

VAP: Ventilator-associated pneumonia
ICU: Intensive care unit
LDH: Lactate dehydrogenase
MIMIC: The Multiparameter Intelligent Monitoring in Intensive Care
SBP: Systolic blood pressure
DBP: Diastolic blood pressure
SPO_2_: Saturation of peripheral oxygen
WBC: White blood cell
INR: International normalized ratio
SOFA: Sequential Organ Failure Assessment
SAPS II: Simplified Acute Physiology Score II
GCS: Glasgow Coma Score
CCI: Charlson comorbidity index
OR: Odds ratio
CI: Confidence interval
RCS: Restricted cubic spline
