# Supplementary material for: Association between lactate dehydrogenase and ventilator-associated pneumonia risk: an analysis of the MIMIC database 2001–2019
Source: BMC Pulm Med. 2024 Jun 6;24:273. doi: 10.1186/s12890-024-03084-9 (PMC11157856; doi:10.1186/s12890-024-03084-9)
Supplement: Supplementary file 1 — Supplementary Material 1 [file 12890_2024_3084_MOESM1_ESM.docx]

**Supplementary Table S1**. Screening for confounders associated with ventilator-associated pneumonia (VAP) risk using univariable logistic regression model

| **Variables** | **OR (95%CI)** | ***P*** |
| --- | --- | --- |
| Age | 0.827 (0.765-0.893) | <0.001 |
| Gender |  |  |
| Female | Ref | - |
| Male | 1.527 (1.290-1.806) | <0.001 |
| Insurance |  |  |
| Medicare | Ref | - |
| Others | 1.251 (1.065-1.470) | 0.006 |
| Sepsis |  |  |
| No | Ref | - |
| Yes | 1.874 (1.591-2.209) | <0.001 |
| Weight | 0.944 (0.333-2.675) | 0.913 |
| Heart rate | 1.027 (0.949-1.112) | 0.508 |
| SBP | 1.050 (0.970-1.137) | 0.227 |
| DBP | 1.103 (1.021-1.192) | 0.012 |
| Respiratory rate | 0.980 (0.904-1.062) | 0.623 |
| Temperature | 1.042 (0.961-1.130) | 0.319 |
| Spo2 | 0.952 (0.884-1.024) | 0.186 |
| SOFA | 1.198 (1.113-1.289) | <0.001 |
| SAPSII | 1.196 (1.108-1.292) | <0.001 |
| GCS | 1.099 (1.001-1.208) | 0.048 |
| CCI | 0.903 (0.831-0.980) | 0.015 |
| WBC | 0.983 (0.902-1.071) | 0.697 |
| INR | 0.978 (0.897-1.065) | 0.606 |
| PT | 1.016 (0.941-1.096) | 0.688 |
| Ventilation |  |  |
| Invasive mechanical ventilation | Ref | - |
| Non-invasive mechanical ventilation | 0.221 (0.181-0.269) | <0.001 |
| Ventilation duration hours | 1.460 (1.380-1.544) | <0.001 |
| Vasopressors |  |  |
| No | Ref | - |
| Yes | 1.618 (1.375-1.903) | <0.001 |
| Antibiotics |  |  |
| No | Ref | - |
| Yes | 1.274 (1.045-1.553) | 0.016 |
| Myocardial infarct |  |  |
| No | Ref | - |
| Yes | 0.879 (0.711-1.086) | 0.232 |
| Congestive heart failure |  |  |
| No | Ref | - |
| Yes | 0.942 (0.795-1.116) | 0.490 |
| Liver disease |  |  |
| No | Ref | - |
| Yes | 1.406 (1.168-1.692) | <0.001 |
| Malignant cancer |  |  |
| No | Ref | - |
| Yes | 0.585 (0.446-0.766) | <0.001 |
| Pneumonia |  |  |
| No | Ref | - |
| Yes | 0.861 (0.714-1.039) | 0.119 |
| COPD |  |  |
| No | Ref | - |
| Yes | 0.955 (0.784-1.162) | 0.645 |
| AKI |  |  |
| No | Ref | - |
| Yes | 4.640 (3.184-6.761) | <0.001 |
| ARDS |  |  |
| No | Ref | - |
| Yes | 2.006 (1.671-2.409) | <0.001 |

Note: OR, odds ratio; CI, confidence interval; Ref, reference; SBP, systolic blood pressure; DBP, diastolic blood pressure; SPO2, saturation of peripheral oxygen; SOFA, Sequential Organ Failure Assessment score; SAPSII, Simplified Acute Physiology Score Ⅱ; GCS, Glasgow Coma Score; CCI, Charlson comorbidity index, WBC, white blood cell; INR, international normalized ratio; PT, prothrombin time; COPD, chronic obstructive pulmonary disease; AKI, acute kidney injury; ARDS, acute respiratory distress syndrome.

**Supplementary Table S2**. Effects of LDH and confounders on ventilator-associated pneumonia (VAP) (model 3).

| **Variables** | **LDH (continuous)** | | **LDH (categorical)** | |
| --- | --- | --- | --- | --- |
|  | **OR (95%CI)** | ***P*** | **OR (95%CI)** | ***P*** |
| LDH (continuous) | 1.15 (1.06-1.24) | <0.001 |  |  |
| LDH (categorical) |  |  |  |  |
| <210 IU/L |  |  | Ref | - |
| 210-279 IU/L |  |  | 1.09 (0.84-1.40) | 0.520 |
| 279-390 IU/L |  |  | 1.38 (1.08-1.76) | 0.010 |
| >390 IU/L |  |  | 1.50 (1.18-1.90) | <0.001 |
| Age | 0.79 (0.72-0.87) | <0.001 | 0.79 (0.72-0.87) | <0.001 |
| Gender |  |  |  |  |
| Female | Ref | - | Ref | - |
| Male | 1.44 (1.21-1.71) | <0.001 | 1.44 (1.22-1.71) | <0.001 |
| Sepsis |  |  |  |  |
| No | Ref | - | Ref | - |
| Yes | 1.57 (1.33-1.87) | <0.001 | 1.57 (1.32-1.86) | <0.001 |
| SOFA | 1.00 (0.92-1.09) | 0.979 | 1.00 (0.92-1.09) | 0.990 |
| SAPSII | 1.22 (1.10-1.34) | <0.001 | 1.22 (1.11-1.35) | <0.001 |
| GCS | 1.15 (1.05-1.26) | 0.004 | 1.15 (1.05-1.26) | 0.004 |
| CCI | 0.99 (0.89-1.09) | 0.785 | 0.98 (0.89-1.09) | 0.757 |
| Liver disease |  |  |  |  |
| No | Ref | - | Ref | - |
| Yes | 1.11 (0.90-1.36) | 0.336 | 1.11 (0.90-1.36) | 0.327 |
| Malignant cancer |  |  |  |  |
| No | Ref | - | Ref | - |
| Yes | 0.56 (0.42-0.76) | <0.001 | 0.56 (0.42-0.76) | <0.001 |
| AKI |  |  |  |  |
| No | Ref | - | Ref | - |
| Yes | 4.10 (2.79-6.02) | <0.001 | 4.10 (2.79-6.01) | <0.001 |
| ARDS |  |  |  |  |
| No | Ref | - | Ref | - |
| Yes | 1.43 (1.17-1.75) | <0.001 | 1.44 (1.17-1.76) | <0.001 |

Note: OR, odds ratio; CI, confidence interval; Ref, reference; LDH, lactate dehydrogenase; SOFA, Sequential Organ Failure Assessment score; SAPSII, Simplified Acute Physiology Score Ⅱ; GCS, Glasgow Coma Score; CCI, Charlson comorbidity index, AKI, acute kidney injury; ARDS, acute respiratory distress syndrome.

**Supplementary Table S3**. Effects of LDH and confounders on duration of mechanical ventilation (model 3).

| **Variables** | **LDH (continuous)** | | **LDH (categorical)** | |
| --- | --- | --- | --- | --- |
|  | **Beta (95%CI)** | ***P*** | **Beta (95%CI)** | ***P*** |
| LDH (continuous) | 4.49 (3.42, 5.56) | <0.001 |  |  |
| LDH (categorical) |  |  |  |  |
| <210 IU/L |  |  | Ref | - |
| 210-279 IU/L |  |  | 1.21 (-1.76, 4.17) | 0.425 |
| 279-390 IU/L |  |  | 3.84 (0.86, 6.82) | 0.011 |
| >390 IU/L |  |  | 11.22 (8.21, 14.22) | <0.001 |
| Age | -7.49 (-8.73, -6.24) | <0.001 | -7.54 (-8.78, -6.29) | <0.001 |
| Gender |  |  |  |  |
| Female | Ref | - | Ref | - |
| Male | 0.68 (-1.45, 2.80) | 0.533 | 0.71 (-1.42, 2.84) | 0.514 |
| Sepsis |  |  |  |  |
| No | Ref | - | Ref | - |
| Yes | 4.70 (2.30, 7.09) | <0.001 | 4.70 (2.31, 7.10) | <0.001 |
| SOFA | 1.78 (0.56, 3.00) | 0.004 | 1.79 (0.57, 3.01) | 0.004 |
| SAPSII | 8.65 (7.27, 10.04) | <0.001 | 8.72 (7.34, 10.11) | <0.001 |
| GCS | 3.91 (2.79, 5.03) | <0.001 | 3.92 (2.80, 5.04) | <0.001 |
| CCI | 0.28 (-1.00, 1.56) | 0.667 | 0.30 (-0.98, 1.58) | 0.644 |
| Liver disease |  |  |  |  |
| No | Ref | - | Ref | - |
| Yes | -4.16 (-6.99, -1.33) | 0.004 | -4.13 (-6.97, -1.30) | 0.004 |
| Malignant cancer |  |  |  |  |
| No | Ref | - | Ref | - |
| Yes | -4.81 (-8.01, -1.61) | 0.003 | -4.90 (-8.10, -1.69) | 0.003 |
| AKI |  |  |  |  |
| No | Ref | - | Ref | - |
| Yes | 11.77 (8.86, 14.69) | <0.001 | 11.81 (8.89, 14.73) | <0.001 |
| ARDS |  |  |  |  |
| No | Ref | - | Ref | - |
| Yes | 5.34 (2.29, 8.39) | <0.001 | 5.32 (2.27, 8.37) | <0.001 |

Note: OR, odds ratio; CI, confidence interval; Ref, reference; LDH, lactate dehydrogenase; SOFA, Sequential Organ Failure Assessment score; SAPSII, Simplified Acute Physiology Score Ⅱ; GCS, Glasgow Coma Score; CCI, Charlson comorbidity index, AKI, acute kidney injury; ARDS, acute respiratory distress syndrome.


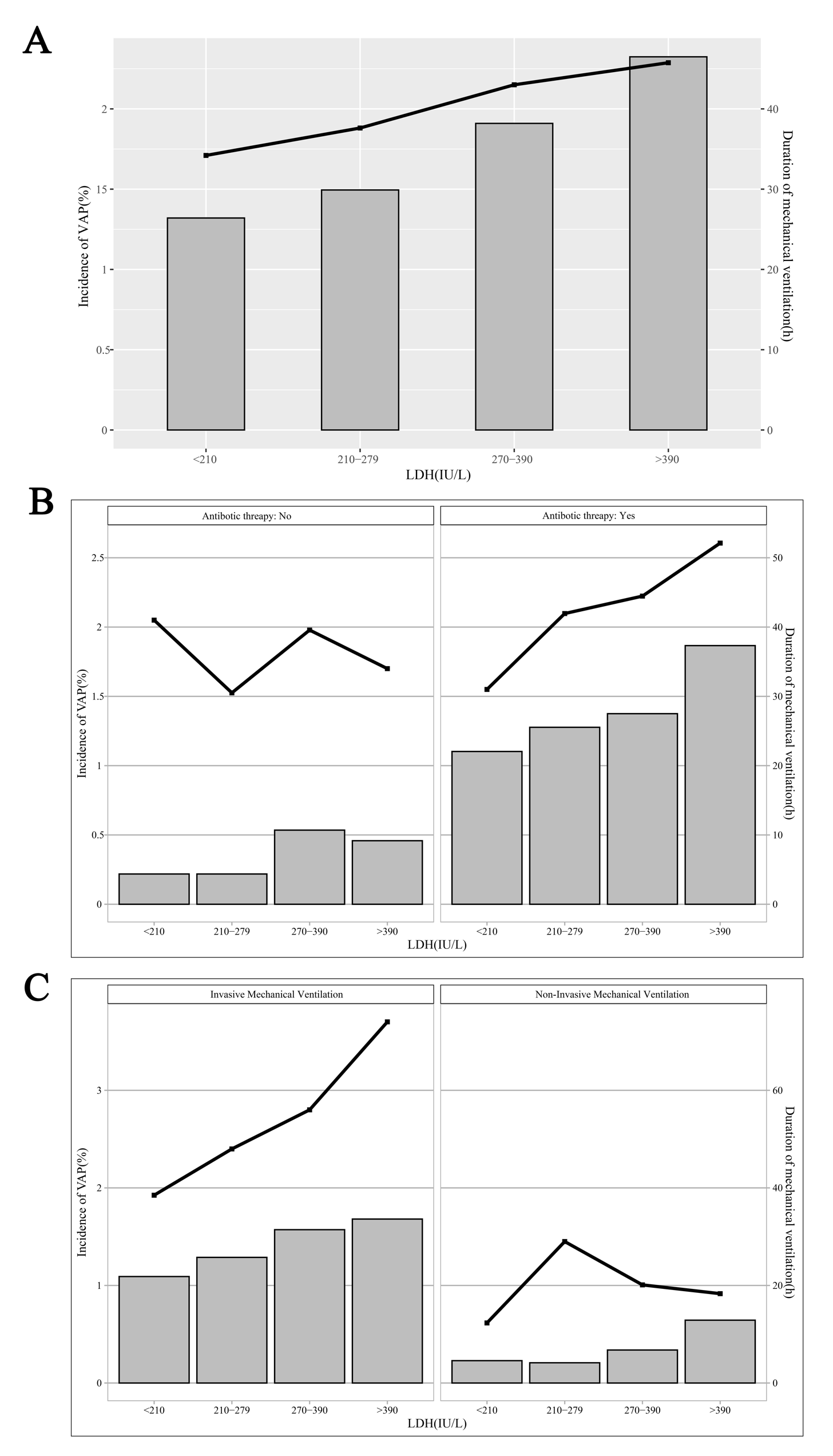


**Supplementary Figure S1**. The incidence of ventilator-associated pneumonia (VAP) and duration of mechanical ventilation in the four lactate dehydrogenase (LDH) level groups. The bar graph is the incidence of VAP (left y-axis) and the line graph is the duration of mechanical ventilation (right y-axis). (A) overall populations; (B) populations with or without antibiotics therapy; (C) populations with invasive or non-invasive mechanical ventilation.
